# Results of brainstem evoked response in patients with vestibular complaints

**DOI:** 10.1590/S1808-86942010000300019

**Published:** 2015-10-20

**Authors:** Gisiane Munaro, Aron Ferreira da Silveira, Angela Garcia Rossi, Daiane Korbes, Andréa Dulor Finkler

**Affiliations:** 1 Audiologist (IPA/IMEC); MSc in Human Communication Disorders (UFSM), Speech and Hearing Therapist; 2 PhD in Experimental Surgery (UFSM), Full Professor of Histology and Embryology in the Veterinary Medicine Program. Head of the Morphology Department (UFSM); 3 PhD in Human Communication Disorders (UNIFESP), Adjunct Professor – Speech and Hearing Therapy Program – Universidade Federal da Santa Maria (UFSM); 4 Audiologist. MSc in Human Communication Disorders (UFSM), Speech and Hearing Therapist; 5 Audiologist and MSc student in Human Communication Disorders (UFSM), Speech and Hearing Therapist

**Keywords:** dizziness, electrophysiology, hearing, vertigo

## Abstract

Otoneurological evaluations are based on tests which investigate auditory and vestibular disorders, including brainstem evoked auditory potentials and vecto-electronystagmography.

**Aim:** to describe the results from the otoneurological assessment of patients with vestibulocochlear complaints, normal hearing individuals and patients with hearing loss, and we will compare them to a control group.

**Materials and Methods:** Cross-sectional, retrospective, observational study, held with 56 dizzy patients assessed by means of audiometry, vecto-electronystagmography and brainstem evoked auditory potential, broken down into Group A, with 31 normal-hearing individuals and Group B with 25 hearing loss patients, compared to the control group made up of ten normal-hearing asymptomatic individuals.

**Results:** Patients from groups A and B were compared to the Control Group, although with values within the normal range. A common finding for both groups was the lack of wave I at 80 dBHL and it happened bilaterally in four individuals (12.9%) and unilaterally in three (9.6%) for Group A; and bilaterally in eight individuals from Group B (32%). In the two cases in which vecto-electronystagmography showed central vestibular alteration, there were no changes to the evoked potential parameters.

**Conclusion:** patients with vertigo, normal-hearing and hearing loss individuals had increased absolute latencies when compared to the Control Group.

## INTRODUCTION

The functional assessment of a patient with vertigo must include an auditory investigation, with audiometry and impedance measures in order to have an audiological profile of the patient. These tests must be done regardless of the patient reporting auditory disorders, because they may show changes which help in the final diagnosis^1^.

The neurotology investigation is based on procedures used for investigating auditory and vestibular disorders in order to obtain information which may contribute to the diagnosis of labyrinthine changes^2^. The neurology test may comprehend a set of tests, including auditory exams aiming at the clinical evaluation of the vestibular apparatus and its association with other organs and systems^3^.

Vector-electronystagmography (VENG) is the most often used method to assess and diagnose vestibular disorders, although it is a long duration test which requires the active participation of the individual being assessed, and it has a certain degree of discomfort. Brainstem Auditory Evoked Potentials (BAEPs) is but an electrophysiological measure with important applications in the differential diagnosis in audiology. These exams are part of the set of tests which allow tracing the patient's neurotological makeup^2^.

BAEPs represent a useful tool insofar as central and peripheral auditory and non-auditory disorders are concerned, especially for being a non-invasive physiological method which helps in the topodiagnosis of auditory lesions. Nonetheless, it is only through concurrent auditory symptoms that this test is indicated for patients with vertigo, in such a way that there is a lack of studies correlating evoked auditory potential findings and vestibular exams in patients with symptoms of unbalance, falls, dizziness or vertigo. Changes to the vestibular and auditory systems seem to trigger investigatory interest, so that clinical alterations may help in the diagnostic process.

Correlations between BAEPs and the vestibulocochlear anatomy show that peripheral vestibular changes did not impact the electrophysiological responses; however, the involvement of the central vestibular areas such as the brainstem, even if by minimum dysfunction – especially vascular, can impact the auditory tracts, changing the evoked potentials. Diseases which are capable of changing the BAEPs include auditory nerve compression, vascular disorders impacting the vestibulocochlear nerve and demyelinating lesions^4^.

The definition of the most anterior waves can be compromised by peripheral diseases near the VIII nerve, in such a way that, as the cochlear loss increases, wave I tends to deteriorate, nonetheless, with the increase in stimulus intensity, possible effects of a peripheral hearing loss are overcame^5^.

There have been studies which reported that among the diseases capable of causing BAEP disorders are the compression of the auditory nerve and that of the brainstem, affecting the vestibulocochlear nerve and demyelinating lesions^6^,^7^,^8^.

The absolute latencies of the BAEP components are affected by factors such as the stimulus intensity, age, gender and auditory status; therefore, these values are not very useful in neurological application as the interpeak latency values, with more consistent information regarding central conduction. BAEPs can also be useful when the patient has nystagmus, which may happen because of peripheral causes such as Ménière's disease, labyrinthitis, neuritis or brainstem lesions, such as multiple sclerosis. Should the nystagmus happen in brainstem lesions, BAEPs can be impaired; however, should the disorder be peripheral, it will be normal^9^.

Inner ear vascularization is an important anatomical correlation between the auditory and vestibular systems, it stems from the vertebrobasilar system from a branch of the basilar trunk, from where the labyrinthine artery arises^10^. the occlusion of the vestibular, labyrinthine or cerebellar arteries may trigger vestibular syndromes^11^. Disorders which result in ischemia of the peripheral and central organs such as postural hypotension, vertebrobasilar atherosclerosis or cervical compression can affect both the auditory and vestibular systems^12^.

The goal of the present study is to observe and describe the characteristics of patients with vertigo symptoms as to the results from the BAEPs, in a group of normal-hearing patients and patients with hearing loss, comparing them to the control group.

## MATERIALS AND METHODS

This is an observational, descriptive, cross-sectional and retrospective study. The study project was registered at SISNEP under # 0045.0.111.000-08 and approved by the Ethics Committee of the institution where it was carried out, under protocol # 261B/08, authorizing the use of the data bank.

The patients whose main complaints were vertigo or dizziness came referred for neurotological assessment in a clinical center between January of 2006 and December of 2008 and underwent auditory, vestibular and BAEP exams. Of the 56 vertigo patients who made up the sample, we had 31 patients with normal auditory thresholds, aged between 15 and 60 years – mean age of 40, in group A; and group B with 25 patients with hearing, aged between 30 and 84 years, with a mean age value of 58 years. The control group was made up of ten normal-hearing individuals without auditory or vestibular complaints, with ages between 18 and 30 years, mean of 26 years, who underwent audiometry and BAEPs.

Vertigo patients were assessed by means of an anamneses, ear inspection, static and dynamic balance tests (Romberg, Romberg-Barré, Unterberger and gait test), cerebellar coordination tests (index-index, index knee-nose and diadochokinesia), Dix-Hallpike, VENG, BAEP and tonal and vocal audiometry.

Individuals with visual, neurological, cognitive changes were taken off the study, as were those who did not undergo tonal audiometry, BAEP and vestibular tests, as well as those with severe and profound hearing loss, considering the mean value of the tonal thresholds in the frequencies of 500, 1,000 and 2,000 Hz or ear asymmetries between the ears with a difference greater than 20 dB, in any frequency, besides conductive or mixed hearing loss. The degree of hearing loss was established with the goal that all the waves should be present for analysis, which is expected for losses up to moderate intensity^13^. The hearing loss degree of group B was estimated in up to 70 dB or moderate.

Tonal threshold audiometry was carried out in a sound-treated booth with the Clinical Audiometer AC 40 and the Diagnostic Audiometer AD 228b, from Interacoustics, calibrated according to the ANSI- 69 standard. We analyzed the frequencies of 250 to 8,000 Hz (air conduction) and 500 to 4,000 Hz (bone conduction), according to the method used to determine the descending-ascending auditory threshold, using the warble tone.

Auditory tests were carried out in order to differentiate the groups and are hereby described in order to rule out the possible hearing loss interference on BAEP interpeak intervals and latencies. The results obtained from the audiometric test were interpreted based on the ISO 1999 standard^14^, which defines the hearing loss standards, with modifications done by the authors of the present study in order to classify the subjects with hearing loss only or those restricted to the low and high frequencies.

The vestibulo-ocular registers with electrodes were carried out with the help of the vector-electronystagmography computerized system – Vecwin version 5.0, from Neurograff, where the patients sat at 1 m from the light bar. VENG involved the observation and recording of eye movements during spontaneous nystagmus with the eyes open and shut, semi-spontaneous nystagmus, pre-caloric nystagmus and oculomotor tests (calibration, saccadic movements, pendular tracking, and optokinetic), decreasing pendular rotational test (DPRT) and caloric tests. Heat stimuli were done using air in the temperatures of 42° and 18° in both ears during 80 seconds, with intervals of 3 minutes between them. In the eyes closed test the patients underwent mental alert tasks in order to maximize vestibulo-ocular reflex responses.

The electrophysiological evaluation was carried out with the Hortmann/BERA modul. v 5.07 equipment, click stimulus and −30dB contralateral masking in relation to the 80 dB HL, with supra-aural Hortmann beyerdynamic DT48 phones. Adhesive electrodes were placed on the mastoid surface and on the forehead after skin cleaning and with conductive paste. The patients remained lying down in a silent and darker environment, with their eyes closed and were instructed to relax or sleep in order to reduce the interference of artifacts. Each ear was tested twice in order to check for wave reproducibility.

Descriptive analysis was held for the data collected during anamneses and for the vestibular and auditory evaluations for groups A and B. These groups were not compared to each other, but only in relation to the control group. Statistical analysis, done with the help of the SAS -Statistical Analysis System 2001, v.9.1.3 software through the ANOVA Variance Analysis, we compared the mean values of the latency and interpeak values per ear between the individuals from both groups assessed by BAEP. The Tukey test allowed us to calculate the mean values and the classification of the differences between groups A and B in relation to the control group. The critical level of significance used was 5%. The results analysis compared the variables studied, which are the absolute latencies of waves I, III and V, wave V interaural difference, I-V and III-V interpeak latencies from groups A, B and control, and they were also compared to the control groups.

## RESULTS

In the anamneses, considering Group A, eleven (35.43%) patients and, in Group B, seven (28%) reported sound health, without a past of diseases or apparent symptoms. 64.52% of the Group A patients and 72% of those from Group B reported one or more vertigo-associated disorders, cervical disorder and high blood pressure -which were the disorders that occurred more frequently in both groups. Results can be seen on Table 1. Auditory and vestibular symptoms are described on Table 2 and may have happened in association, with more than one reference per patient.

**Table 1:** Disorders of symptoms associated to the vestibulocochlear complaints, reported by the patients in the anamneses.

Symptoms	Group A		Group B	
	n	%	n	%
Cervical disorder	9	45,00	4	22,22
Hypertension	4	20,00	10	55,55
Headache	3	15,00	2	11,11
Diabetes	–	–	2	11,11
Depression	3	15,00	1	5,55
Hypercholesterolemia	2	10,00	1	5,55
Memory Disorder	2	10,00	1	5,55
Panic syndrome	1	5,00	–	–
Epilepsy	1	5,00	–	–
Paresis	1	5,00	–	–
Fibromyalgia	–	–	1	5,55
Thyroid changes	–	–	1	5,55
Polyneuropathy	–	–	1	5,55
Méniére's disease	–	–	1	5,55

**Table 2:** Auditory and vestibular symptoms reported by the patients.

Symptoms	Group A		Group B	
	n	%	n	%
Tinnitus	13	41,92	10	40,00
Ear fullness	12	38,74	5	20,00
Non-rotational dizziness	20	64,54	14	56,00
Rotational dizziness	19	61,24	10	40,00
Nausea or vomits	14	45,13	12	48,00
Fall	4	12,92	4	16,00
Body deviation	10	32,22	6	24,00
Unbalance	7	22,51	4	16,00

All Group A patients had auditory thresholds within normal ranges, which worst threshold was 25 dB HL. In Group B, the patients had sensorineural hearing loss with mild to moderate hearing loss – unilateral in six (24%) and bilateral in 19 (76%). On the right ear, 21 (84%) patients had sensorineural hearing loss and three (12%) had normal hearing thresholds. Considering the left ear, 22 (88%) patients had sensorineural hearing loss and three (12%) had normal hearing loss.

The calibration and the saccadic movements were regular in all the patients in the sample. The changes found in the vestibular assessments in Group A patients were asymmetry in the rotational test in one (3.2%) individual, without changes suggesting central vestibular involvement in this group of patients. Concerning Group B, changes such as asymmetrical optokinetic test, type III pendular tracking and bidirectional semi-spontaneous nystagmus were found in two (8%) patients who were diagnosed with central vestibular syndrome, based on the findings hereby described. Both showed normal reflexes in the vestibular test.

In Group A, the caloric test proved normal in nine (29%) individuals, PDN in five (16.1%), unilateral hyperreflexia in ten (32.2%) and bilateral in seven (22.6%). In Group B, ten (40%) individuals had normal reflexes, four (16%) had bilateral hyper-reflexes and two (8%) had unilateral reflex, nine (36%) had PDN. Hyporeflexia and PL did not happen as results in the caloric test of the sample.

In Group A, wave I was bilaterally absent in four individuals and unilaterally absent in the left ear in three cases, making it impossible to analyze interval I-V in four patients in the right ear and in seven patients in the left ear. Waves III and V were present in all the patients evaluated, bilaterally. The group A right ear wave I latency mean values showed highly significant difference in comparison to the control group. In analyzing waves III and V we see a significant difference according to the value of p, depicted on Table 3. On the left ear, wave I showed a highly significant difference, as it happened to the right ear. Wave III presented statistically significant difference, as it happened to the right ear. Wave III showed a statistically significant difference; however, the same did not happen to Wave V in this ear.

**Table 3:** Absolute latency values from Group A and comparison with the Control Group.

	Wave I		Wave III		Wave V	
	RE	LE	RE	LE	RE	LE
Mean A	2,07	2,04	4,14	5,98	6,00	5,98
Standard deviation	0,08	0,06	0,13	0,26	0,16	0,26
TOTAL	27	24	31	31	31	31
Mean-Control	2,00	1,97	4,02	4,01	5,86	5,84
p value	0,0082*	0,0015*	0,0105*	0,0183*	0,0132*	0,1176
F significance	7,86	12,09	7,23	6,07	6,74	2,56

In Group B, wave I was absent in the right ear of ten individuals and in the left ear of nine individuals; and bilateral in eight individuals, making it impossible to analyze the I-V interval in these patients. Waves III and V were present in all patients from Group B bilaterally. The analysis of the waves I, III and V latency mean values in Group B, in comparison with the control Group showed a highly statistically significant difference for both ears, according to Table 4.

**Table 4:** Absolute latency values in Group B and comparison with the control group.

	Wave I		Wave III		Wave V	
	RE	LE	RE	LE	RE	LE
Mean B	2,11	2,07	4,19	4,21	6,09	6,09
Standard deviation	0,07	0,07	0,16	0,18	0,22	0,23
TOTAL	15	16	25	25	25	25
Mean-Control	2,00	1,97	4,02	4,01	5,86	5,84
p value	0,0006*	0,0009*	0,0026*	0,0027*	0,0044*	0,0030*
F significance	15,78	14,48	10,62	10,56	9,35	10,31

For both Groups, the mean values of intervals I-V and III-V, in comparison to the control group, did not show statistically significant difference in both ears, as per the results depicted on Table 5.

**Table 5:** Interpeak values from groups A and B and comparison with the Control Group.

	Group A				Group B			
	I-V		III-V		I-V		III-V	
	RE	LE	RE	LE	RE	LE	RE	LE
Mean sample	3,91	3,94	1,86	1,87	3,95	3,97	3,95	3,97
Mean-control	3,86	3,87	1,84	1,82	3,86	3,87	3,86	3,87
p value	0,3073	0,2174	0,4504	0,3262	0,1891	0,2069	0,1891	0,2069
F significance	1,07	1,58	0,58	0,99	1,83	1,68	1,83	1,68

In the Control Group, the individual analysis per patient in order to check the interaural difference of wave V and I-V and III-V intervals showed that all the waves were present and there were no interaural changes between the waves or interpeaks greater than 0.15 ms. The results of the mean values of latencies in the Control Group are depicted on Tables 3 to 5, together with the results from groups A and B, having in mind that its use was comparative.

In Group A we found interaural variations between the waves and interpeaks of up to 0.25 ms for 30 patients. Only one (3.23%) patient presented an interaural difference on wave V of 1.0 ms. The VENG results showed hyperreflexia at 18° on the left ear, characterizing irritative peripheral vestibular syndrome. Immittance measures showed the presence of acoustic reflexes. This patient returned to the ENT with a request for complementary tests aiming at diagnostic accuracy.

Concerning Group B, the interaural variations between waves and interpeak intervals are situated in a peak of 0.35 ms for 24 patients. Only one (4%) patient presented an interaural difference in wave V of 0.5 ms and wave III of 0.7 ms. The III-V interpeak difference was 1.7 ms. The aforementioned patient was diagnosed as having Ménière's disease for 10 years, her vestibular test showed PDN and irritative peripheral syndrome as a result. Because of BAEP results, the speech and hearing therapist's opinion was inconclusive, and a complementary evaluation was required after referral to the otorhinolaryngologist.

## DISCUSSION

In the present study, 64.52% of Group A patients and 72% from Group B reported one or more disorders associated to vertigo, being cervical disorder and arterial hypertension the ones occurring more often in both groups. A study^15^ calls attention to cervical disorder as one of the main extravestibular causes of vertigo. Among them we have heart diseases, hypertension, atherosclerosis, cerebral ischemia, renal insufficiency, diabetes mellitus and thyroid disorders^16^.

Over 300 clinical manifestations can be found among patients with vestibulocochlear symptoms such as dizziness, vertigo, unbalance, falls, syncope, nausea, vomit, tinnitus, deafness and hypersensitivity to sound. The most common diseases in which tinnitus can be associated to vertigo are Ménière's disease, metabolic labyrinthine disorders, vestibular migraine, vascular vestibular diseases, post-traumatic vertigo, vertebrobasilar insufficiency, presbyvertigo, presbycusis, and others^17^.

Tinnitus was present in 23 (41.07%) of the patients in the sample and the feeling of stuffed ear in 17 (30.35%) patients with a greater occurrence of both symptoms for Group A. Vertigo followed by unilateral auditory hearing loss, tinnitus or a feeling of stuffed ear, when starting together, strongly suggest a peripheral cause of vestibular disorders^12^. The association of tinnitus and vertigo is a common finding in vestibular disorders such as Ménière, metabolic labyrinthine diseases, vestibular migraine, vascular-vestibular disorders, post-traumatic vertigo, vertebrobasilar insufficiency, presbyvertigo, presbycusis, and others^17^.

Two patients from Group B with central findings upon the VENG showed normal reflex in the caloric test. BAEP presented normal results in one of these cases and presented increased latencies in the other case for waves III and V in 4.6 and 6.7 ms, respectively. The I-V interval was not assessed by the absence of Wave I in both individuals. BAEP can be normal in cases of central origin disorder if the auditory pathway is not involved, including changes in the brainstem, as it happens in degenerative and demyelinating disorders or in vascular impairment of the brainstem restricted to the ventral area 9.

In vertigo patients with tinnitus and many neurotological disorders such as Ménière, vestibular schwannoma, BPPV, vestibular neuritis, sudden hearing loss and trauma-related vertigo, ENG was the best test used to differentiate central from peripheral involvement18. In a study held, BAEP was altered in patients with vascular-vertigo, without wave I, increased absolute V wave, I-V and I-III interpeak increase. The vestibular test was altered in all the patients. Thus, the two methods proved efficient to help in the diagnosis of vertigo^19^.

In a study, the diagnostic value of BAEP in vertigo was considered positive, with changes found in 18% of the cases, with an increase in the III-V interval latency and no waves III or V, results which, although altered, are different from the findings in our series^20^. All patients with disorders showed evidence of an associated organic disorder, although the cause is not established for most of the cases – which is common for patients with dizziness.

In the present study, the individuals who make up groups A and B had normal BAEP values in 96% of the cases; nonetheless, when compared to the Control Group, there was a single-block increase in absolute latencies. The lack of wave I was a finding in both groups: normal hearing and those with hearing loss of up to moderate level. We did not find clear references in the literature about this finding in patients with normal psychoacoustic thresholds. However, it is inferred that, when auditory thresholds allow wave I to be present and it does not show up, one can think of a disease involving this region, suppressing its visualization.

In a study involving normal-hearing individuals, the authors concluded that the wave I extension and other BAEP wave delays are compatible with peripheral auditory lesions and can reflect hearing loss in ultra-high frequen-cies^21^ – which were not assessed in the present study.

In sensorineural hearing loss there can be an extension of wave I and its subsequent components, or reduction of the I-V interval, as well as wave I being absent, according to the level of loss. The latency extension can happen because of an increase in wave I latency as a result of an auditory loss on the high frequencies, not identified in the tonal audiometry from 250 to 8,000 Hz; however, the interpeaks are not very much affected in function of the hearing loss and, even in these cases, BAEP can be reliably used in order to establish the function involving the central auditory pathways^9^.

One of the few studies concerning the lack of wave I was carried out with adult individuals who were audiologically normal and in whom we did not observe waves I and III at 80 dB HL in the frequencies of 500; 1,000; 2,000 and 4,000 Hz, in the BAEP with a tone burst stimulus^22^.

The increase in absolute latencies with unaltered interpeak intervals is a common finding in conductive hearing loss^23^,^9^ and it was also found in patients with Ménière^24^.

Our findings on the latency increase are very similar to the studies which BAEP results showed significant increase in waves I, III and V latencies in 43% of the normal hearing patients complaining of tinnitus when compared to the Control Group, as well as interpeak values within normal ranges^21^.

The present investigation was also similar to latency increase findings from waves I, III and V in patients with vertigo alone or vertigo associated with tinnitus. Moreover, the authors also noticed a delay in I-III and III-V interpeak latencies^25^, which was not seen in the present study.

There was a study in which patients with tinnitus but not vertigo or hearing loss, were compared to a control group. Changes were seen in 47% of the individuals with waves I, III and V wave latencies extension, besides an increased III-V^26^ interpeak, getting close to the results from the present study.

It may be that BAEP is a highly sensitive test to differentiate cochlear or retrocochlear changes, although its specificity is not very high^24^, diffuse or minimum changes which alter neural synchrony could result in an increase in absolute latencies. The use of higher intensities is useful in order to visualize all the components, in such a way that this could have been increased to higher than 80 dB HL when wave I went missing. The change in click characteristics and on polarity patterns may sensitize the test, helping in the topographic diagnosis of the hearing loss, especially in retrocochlear disorders^27^. Moreover, the fact that the patients in the sample had hearing complaints associated to many disorders, even with normal hearing, may have become an influence factor on the results.

## CONCLUSION

Normal hearing individuals with vestibulocochlear complaints and patients with hearing loss had increased absolute latencies in the BAEP test and complete absence of wave I when compared to normal hearing individuals without vestibular or auditory complaints. These changes corroborate the diagnosis of vestibular involvement, even if its pathophysiology is not well explained. Additional studies must be carried out under different circumstances in order to go more in depth regarding the findings of the present study.
